# Entrainment of a thalamocortical neuron to periodic sensorimotor signals

**DOI:** 10.1186/1471-2202-12-S1-P135

**Published:** 2011-07-18

**Authors:** Dennis Guang Yang, Yixin Guo

**Affiliations:** 1 Departmentof Mathematics, Drexel University, Philadelphia, PA 19104, USA

## 

**Figure 1:**
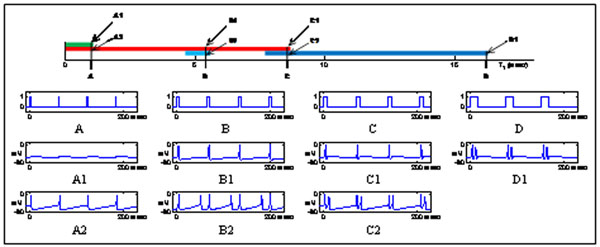
The top panel shows the T_1_ intervals (with T_2_ = 60 milliseconds) for the existence of different families of entrained periodic oscillations (green for sub-threshold oscillations, red for 1-spike oscillations, dark blue for 2-spike oscillations, and light blue for 2-spike oscillations with a delayed second spike.) Panels A, B, C, and D illustrate the periodic excitatory signals with T_2_ = 60 milliseconds and T_1_ = 1.014, 5.399, 8.545, and 16.15 milliseconds, respectively. Panels A1, A2, B1, B2, C1, C2, and D1 show the time series plots of the different types of periodic oscillations entrained to the excitatory signals with T_1_ values indicated in the top panel.

In this work we study the dynamics of a 3-dimensional conductance-based model of a single thalamocortical (TC) neuron in response to sensorimotor signals. In particular, we focus on the entrainment of the system to a periodic excitatory signal that alternates between ‘on’ and ‘off’ states lasting for time T_1_ and T_2_, respectively. By exploiting invariant sets of the system and their associated invariant fiber bundles that foliate the phase space, we reduce the dynamics to the composition of two 2-dimensional maps, with the two components of one of the maps being simply a uniform shift and a uniform decay. With this reduction in computational complexity, we are able to analyze the model’s response to the excitatory signal while varying T_1_ and T_2_ systematically. We find that for fixed T_2_ but different T_1_ there exist and in some cases co-exist entrained periodic oscillations with different number of spikes (see Figure 1 for the case with T_2_ = 60 milliseconds). For relatively large T_2_ (above 55 milliseconds) it is also possible that the model responds to the excitatory signal with delayed spikes. Furthermore, we find that the size of the T_1_ intervals that allow coexistence of different types of entrained oscillations becomes larger as T_2_ increases.
